# Using Matching Traits to Study the Impacts of Land-Use Intensification on Plant–Pollinator Interactions in European Grasslands: A Review

**DOI:** 10.3390/insects12080680

**Published:** 2021-07-28

**Authors:** Jérémie Goulnik, Sylvain Plantureux, Isabelle Dajoz, Alice Michelot-Antalik

**Affiliations:** 1 LAE, Université de Lorraine, INRAE, F-54000 Nancy, France; jeremiegoulnik@gmail.com (J.G.); sylvain.plantureux@univ-lorraine.fr (S.P.); 2 Association Noé, 47 rue Clisson, 75013 Paris, France; 3 Institut d’Ecologie et des Sciences de l’Environnement de Paris, Sorbonne-Université, Université de Paris, 4 Place Jussieu, 75005 Paris, France; isabelle.dajoz@univ-paris-diderot.fr

**Keywords:** pollination function, grasslands, agricultural practices, functional trait, effect trait, plant–pollinator interaction network, floral traits, pollinating insect traits

## Abstract

**Simple Summary:**

Permanent grasslands are main habitats for many plant species and pollinators. Their destruction as well as their intensification has a major impact on plant and pollinator biodiversity, which has a cascading effect on pollination. However, we lack an understanding of these effects, thereby limiting our ability to predict them. In this review, we synthesised the literature on the mechanisms behind this cascade to provide new insights into the relationship between land-use intensification and pollination. By matching functional traits that mediate the relationship between the two trophic levels, we identified major knowledge gaps about how land-use intensification affects plant–pollinator interactions and how it favours plants with generalised floral traits, which are likely harmful to pollination.

**Abstract:**

Permanent grasslands are suitable habitats for many plant and animal species, among which are pollinating insects that provide a wide range of ecosystem services. A global crisis in pollination ecosystem service has been highlighted in recent decades, partly the result of land-use intensification. At the grassland scale, however, the underlying mechanisms of land-use intensification that affect plant–pollinator interactions and pollination remain understudied. In this review, we first synthesise the literature to provide new insights into the relationships between land-use intensification and pollination by using matching community and interaction traits. We then identify knowledge gaps and summarise how land-use intensification of grassland influences floral traits that may in turn be associated with modifications to pollinator matching traits. Last, we summarise how these modifications may affect pollination function on permanent grasslands. Overall, land-use intensification may lead to a shift in flower colour, a decrease in mean nectar tube depth and a decrease in reward production and pollen quality at the community level. This, in turn, may generate a decrease in pollinator mouthparts length and body size, that may favour pollinators that require a low amount of floral reward. We found no study citing the effect of land-use intensification on volatile organic compounds emitted by flowers despite the importance of these molecules in pollinator community composition. Overall, our review highlighted major knowledge gaps about the effects of land-use intensification on plant–pollinator interactions, and suggests that land-use intensification could favour plants with generalised floral traits that adversely affect pollination.

## 1. Introduction

Permanent grasslands cover ca. one-third of Europe’s agricultural area and provide a wide range of ecosystem services [1], but they are in decline as 10 million ha have been lost during the last 50 years [2]. Grasses belonging to the Poaceae family account for most of the plant biomass production on European grasslands, but other plant taxa are of high importance for conservation purposes as well as fodder [3]. In temperate ecosystems, about 78% of angiosperm species depend on pollinators to ensure their sexual reproduction [4]. Global pollinator decline has been observed for the last decades [5,6,7] even though not all species are facing a decline [8], and further robust data are still needed [9]. On grasslands, pollinators play a major role in plant community assembly and in return receive suitable habitats that provide nesting sites and food [6]. Their absence or decline will lead to a decline in the diversity and abundance of plant species [10,11,12].

Insect pollination on permanent grasslands relies on interactions between flowers and pollinators (hereafter, “plant–pollinator interactions”). An approach that includes the morphological, physiological and phenological features of organisms that affect their fitness [13] is useful because plant and pollinator features together drive plant–pollinator interactions. These functional features, called “matching traits” [14] mediate relationships between the two trophic levels [15]. Several plant traits (hereafter, “floral traits”) and pollinator-matching traits are involved in plant–pollinator interactions (Table 1). For example, flowers with deep corollas can only be accessed by pollinators with long mouthparts. Matching trait values can be calculated at the community scale, and the community weighted mean (CWM) is the mean value of traits weighted by the abundance of each species in a community. Functional diversity (FD) is the value, range, and relative abundance of functional traits in a given community [16]. In the mass-ratio hypothesis, an ecosystem’s functions depend on the CWM [16]. The hypothesis of niche complementarity suggests that greater FD values increase niche partitioning and lead to species complementary, which serves the ecosystem functions [17]. These hypotheses have been extensively tested for vegetative functional traits but much less so for the relationships between floral traits and pollination.

On grasslands, floral communities are influenced by agricultural practices. To increase grassland primary production [2], farmers increase defoliation by, for example, defoliating earlier, mowing more frequently, and extending the duration of pasture or density of livestock. They also increase soil nutrient availability through nitrogen fertilisation. The values of these parameters describe the land-use intensity of a grassland. In this review, we focus on increasing land-use intensity (hereafter, “intensification”), as intensification acts as an environmental filter that determines plant community assembly [18]. Intensification selects plant species based on their response traits, defined as traits associated with the response of organisms to variations in environmental factors [19].Little is known about the cascading effects of these changes to the plant community assembly on floral traits [20], which can also be modified by intensification. Floral traits can become more generalised in harsh and frequently disturbed environments, which means that plants have to rely on opportunistic interactions with pollinators. This trait generalisation leads to ecological generalization; [21] that is, a shift from specialist to generalist flowers that are exploitable by most types of pollinators. This has been recorded along different environmental gradients, such as altitude [22] and, to a lesser extent, urbanisation [23], but has only been suggested along intensification gradients on grasslands [24].

The generalisation of floral traits mainly affects the accessibility of floral rewards (pollen, nectar) for pollinators. However, it may also reflect a decrease in the quality of these rewards since many plants favoured by intensification belong to the family Asteraceae [25], which often has low pollen quality [26]. Intensification may also decrease rewards production on grasslands by reducing in the total plant cover that produces rewards and by favouring wind-pollinated grasses [27]. Overall, these changes could have a major impact on pollination, as decreases in floral rewards quantity and quality is a major threat to pollinators [28].

Intensification could also change pollinator community composition. First, total abundance of pollinators, which provides quantitative information about pollination, may fall due to the lower (reward) attractiveness of the grassland and lower food availability. Second, intensification is expected to lead to a decrease in the mean values of pollination effect traits [20], which provide information about the effects of organisms on ecosystem functions [19] (i.e., qualitative information about pollination). Even though traits such as pollinator body size have not been found to be relevant in all ecosystems [29], they are essential to understanding the qualitative differences among pollinators both mechanistically and functionally [30]. Here, we aim to consider both quantitative and qualitative components of pollination because they are rarely considered together despite their high complementarity [14].

We focused on European grasslands under temperate conditions due to the lack of research on other climatic conditions. Using a functional approach, we analysed how grassland intensification affects plant–pollinator interactions and consequently pollination. This review synthesises the literature to identify knowledge gaps, and provide new insights and research avenues into the relationships between intensification and pollination. Our approach is innovative because it is focused on matching traits at the community level. Here we integrate current knowledge and our own assumptions to answer three main questions:

(1) Does grassland intensification lead to more generalised floral traits and a decrease in flower reward production?
(2) Can changes in floral traits generate shifts in pollinator community composition?
(3) Can shifts in pollinator community composition affect pollination function on grasslands, both quantitatively and qualitatively?

## 2. Materials and Methods

The literature survey was conducted using Google Scholar and Web of Sciences between January 2017 and January 2019 for peer-reviewed international articles and PhD theses without a publication date limit. First, we assessed studies that investigated relationships between intensification and floral traits using different combinations of search terms: “land-use intensification”, “land-use change”, “grassland management”, “mowing”, “grazing”, “fertilization” and “flower”, “floral trait”, “flower trait”, “flower colour”, “flower color”, “flower scent”, “nectar tube”, “pollen”, “plant matching trait”, “plant response trait”, “plant effect trait”. The selection process was performed for each floral trait. Then, we assessed studies that investigated relationships between intensification or floral traits and pollinator matching traits or pollination function, adding different combinations of search terms: “pollinator trait/matching trait/body size/effect trait/ response trait”, “pollination”, “plant–pollinator network”, “plant–animal interaction”, “flower visitation”, “interaction frequency”, “pollination function”, “pollen load”, “pollinator effectiveness”, “hairiness”.

We selected publications that corresponded to the following criteria: experimentations on European permanent grasslands except when no study was done in Europe, quantitative measurement or qualitative classification of at least one floral trait or pollinator trait, and direct or indirect relationships between floral traits or pollinator traits or pollination function and land-use index or agricultural practices. We found 13 publications that summarised known and potential effects of intensification on the floral, matching and effect traits of pollinators. These are presented in Table 1. We supplemented this set of publications with articles from other regions or ecosystems.

## 3. Effects of Intensification on Plant–Pollinator Matching Traits

**Figure 1:**
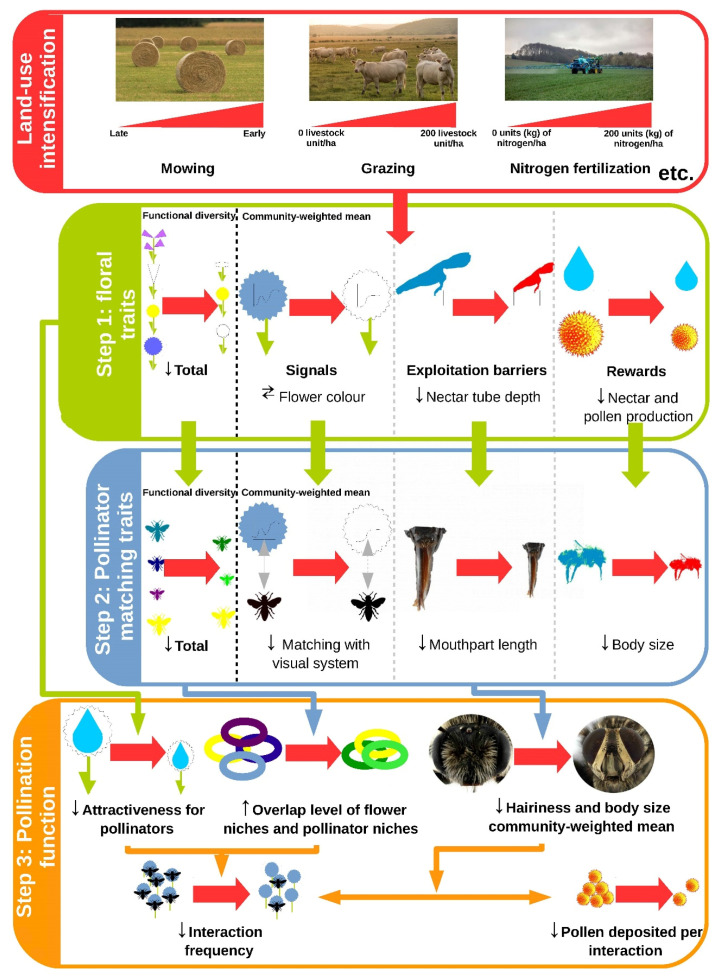
Examples of theoretical cascading effects from land-use intensification to pollination function. The diagram shows only certain expected relationships, but not all were tested. The thick red arrows represent the potential direct or indirect effects of agricultural intensification. The medium-sized colored arrows represent the effects between boxes or within a box (i.e., between steps or within a step in the case of the pollination function). The thin black arrows represent the direction of the expected relationships (upward arrow: increase; downward arrow: decrease; left or right arrow: shift). Agricultural intensification, represented by the red box, combines different parameters (defoliation earliness, duration of pasture or density of livestock, nitrogen fertilization) and could have an effect in step 1 on floral traits (green box). A distinction was made between the three main categories of traits involved in plant–pollinator interactions (signal, exploitation barrier, rewards). In addition, the effects on the total functional diversity and the community weighted mean of traits are distinguished. These influences could have a cascading effect on pollinator matching traits in step 2 (blue box). A distinction was made between functional diversity and community weighted mean of traits but also between pollinator matching traits corresponding to signal, exploitation barrier and rewards. These cascading effects could have an impact on pollination function on the grasslands in step 3 (orange box). Two components of the pollination function are distinguished: the quantitative component with the frequency of plant–pollinator interactions and the qualitative component, represented by the quantity of pollen deposited per interaction. The frequency of interactions can decrease with a decrease in the attractiveness of the grassland due to an overall decrease in floral rewards, or with an increase in niche overlap due to a decrease in the functional diversity of floral traits and pollinator matching traits. The potential decrease in community weighted mean of pollinator hairiness and body size with intensification could generate a decrease in the amount of pollen deposited per interaction. However, the influence of changing composition of the underlying pollinator community could also influence the frequency of interactions.

Figure 1 illustrates the cascading effects from land-use intensification on pollination highlighted in this review. It shows that land-use intensification influenced the FD and CWM of floral traits (step 1), which in turn influenced the FD and CWM of pollinator matching traits (step 2), thereby affecting both quantitative and qualitative components of pollination (step 3).

### 3.1. Effects of Intensification on Pollination Signals

#### 3.1.1. Flower Colour

Intensification can be associated with (i) a shift in the dominant colour of flowers (as perceived by *Apis mellifera*) from blue or yellow to white at the community level and (ii) a decrease in flower colour diversity, but only when measured before the first mowing as recorded in two German regions, [34]. Phylogenetic clustering does not explain this result despite a relationship between flower colour and phylogeny [34]. Pollinators prefer certain colours, due in part to their photoreceptors [51]. For instance, Diptera may be more abundant on grassland plots with either yellow or white flowers, depending on their preferences [43]. As the visual spectrum of insects often extends into the ultraviolet, most pollinators can detect white [52]. Pollinators can also learn to detect other colours, even though the limited learning capacities of Diptera can restrict their shifts toward a different colour [33]. Overall, even though intensification may lead to a higher relative abundance of white flowers, [35] it suggests a matching disruption between flower colour and the visual system of pollinators when intensification is high. Hence, the influence of flower colour on pollinator assemblage remains unclear.

#### 3.1.2. Flower Odours

By modifying plant communities, intensification affects grassland odourscapes, which are assemblages of volatile organic compounds (VOCs) emitted by plants, and in turn alters plant–pollinator interactions [53] and the structure of pollination networks [54]. The only experiment (to the best of our knowledge) that tests this hypothesis [36] found no relationship between botanical composition at the community level after intensification and VOCs. However, this approach was based on only two French mountain grasslands with contrasting degrees of intensification. As for flower colour, pollinators have innate and learned preferences for specific flower scents [55]. For example, [36] found a positive correlation between the relative abundance of benzaldehyde and bumblebee species richness and between the relative abundance of butyrolactone and bee species richness. Furthermore, [54], in the Northern Rocky Mountains of the U.S., showed that the richness of bees (standardized by subnetwork size) increased with floral VOC richness and declined with VOC originality.

However, little information is available on relationships between pollinator matching traits and flower odour traits. Two traits influence a pollinator’s ability to recognise scents: the length of the antennae that bears odorant sensilla and the number of odorant receptor types [55]. For instance, longer antennae may have more receptors, which would increase the ability to detect odours and rely on odour signals or cues to interact with flowers [56]. However, these traits do not provide clues about the flower scent preferences of pollinators. Hence, future studies into the influence of grassland intensification on the relationship between odourscape and pollinator attraction are needed.

### 3.2. Effects of Intensification on Barriers to the Exploitation of Floral Rewards

#### Nectar Tube Depth

**Figure 2:**
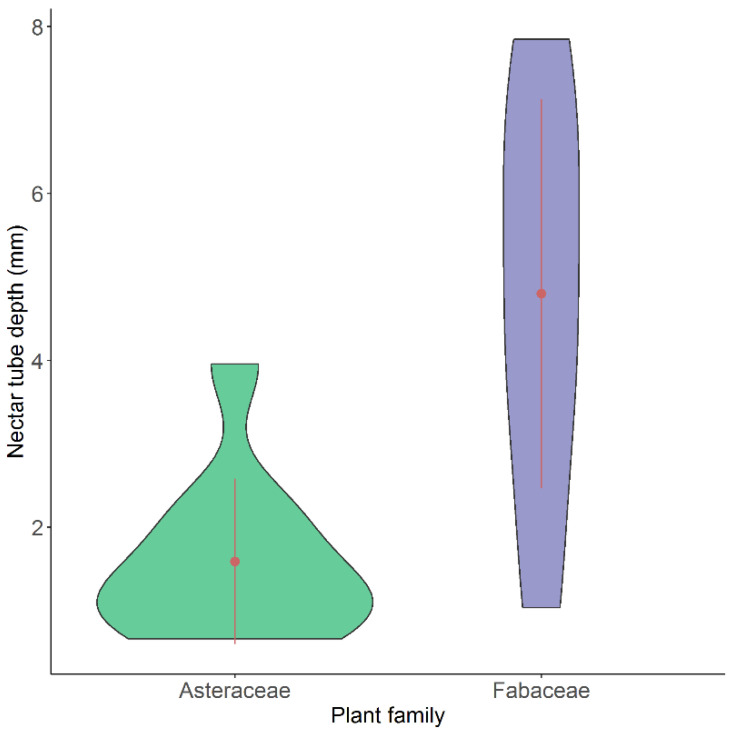
Probability density plots, mean (red point) and standard deviation (red lines) of nectar tube depth (mm) by plant family (Asteraceae or Fabaceae) (10 species per family, 10 individuals per species). Fabaceae have significantly deeper nectar tubes.

The relationship between nectar tube depth and intensification have not been tested at the community level on grasslands. However, existing studies suggest a decrease in mean nectar tube depth with intensification because of a shift in plant community composition. For instance, intensification disadvantages plants from the family Fabaceae, which often have deep nectar tubes [20,27]. [39] found that cover by Fabaceae shifted from 40% of total cover in extensive plots to 10% in intensive plots. Intensification can also favour plants from the families Apiaceae and Asteraceae. On temperate grasslands, species from these two families often have flowers with shallow corollas and easily accessible flower rewards [24,39]. Using our own database, which contains 10 Fabaceae and 10 Asteraceae plant species (10 individuals measured per species) sampled on 16 grasslands in Moselle, France (see [57] for details), and following the same methods as [38] to measure nectar tube depth, we found that Fabaceae had deeper nectar tubes than did Asteraceae (*t*-test: t = −4.00, df = 12.14, *p* = 0.0017; Figure 2). However, additional measurements of nectar tube depth in European grassland plants are needed to validate these results for a larger species pool.

One may expect that the mouthparts of pollinators would decrease along with the mean nectar tube length due to competitive exclusion; that is, pollinators with more mismatch between their mouthparts length and nectar tube depth are outperformed by those with less mismatch, due to longer handling time [26]; however, this trend was not observed by [58], who only highlighted a strong increase in handling time when the mouthparts length was shorter than the nectar tube depth. [40] found a 64% decrease in the CWM of the relative mouthpart length of pollinators with intensification. This decrease may be related to an increase in the relative abundance of Diptera, which on average has a shorter mouthpart than Hymenoptera. Hence, with intensification, the decrease in CWM mouthpart length may match the decrease in nectar tube depth.

Furthermore, a decrease in mean nectar tube depth may lead to an increase in nectar viscosity because in shallow flowers more nectar exposed to air, leading to more evaporation and thus to an increase in carbohydrate concentration [48]. Hence, pollinators that are able to collect viscous nectar, such as most bees [48] and Diptera [43,49] due to their mouthparts anatomy, could be more abundant on intensively managed grasslands.

### 3.3. Effects of Intensification on Rewards

#### 3.3.1. Nectar and Pollen Production

Intensification may lead to a decline in nectar and pollen production on grasslands even though this has not been observed directly. Indeed, this could be due to shifts in plant community composition from animal-pollinated species to wind-pollinated taxa [27]. This was observed in [45] when grazing increased and in [59] when soil nitrogen and phosphorus increased. Concerning pollen production, several results support this hypothesis as they show a positive correlation between pollen volume per flower and the size of the floral display [25] and a decrease in the floral display CWM with intensification [46]. However, concerning nectar production, [44] observed a 25% increase (kg of sugar/ha/year) in Great Britain from 1998 to 2007, which may have been partly caused by a decrease in atmospheric nitrogen deposition. Plant phenology is also a major issue that influences the temporal supply of nectar and pollen for pollinators. Decreasing intensification can delay flowering, estimated as the CWM onset of flowering in multiple European grasslands [18,45].

A decrease in rewards production may prevent certain pollinators from meeting their high metabolic requirements. [40] observed that the CWM body size of pollinators—which positively correlates with the metabolic rate in arthropods [60]––was twice as large on less intensive grasslands than on the most intensive grasslands. This result is partly explained by an increase in the relative abundance of Diptera, which are on average smaller than bees, according to [39]. One can also expect a decrease in the abundance of pollinators such as large or social bees because they require much more pollen to raise larvae or develop the colony [61] compared to Diptera, which has free-living larvae and a strong ability to store protein to produce eggs [62]. By decreasing plant species richness, intensification may also decrease the temporal stability of flower resources, thus mainly affecting pollinators that need nectar and pollen throughout the season [63]. This is the case for bumblebees, which cannot store large quantities of pollen [26], and also for most social and multivoltine bee species. Pollinators with a short period of activity may also be disadvantaged by intensification if they face a resource shortage when they emerge [64]. To confirm these assumptions, studies are needed on the relationships between intensification and pollinator metabolic requirements on grasslands.

#### 3.3.2. Pollen Quality

Although the nutritional quality of pollen differs greatly among plant species [65] and families [66], no study (to the best of our knowledge) has assessed the effects of intensification on pollen quality at the community level. Plants from the Asteraceae family seem to produce pollen of lower nutritional quality (based on amino-acid composition and protein content; [66]) than do Fabaceae, and intensification promotes the former [39] but disadvantages the latter [20,27]. Hence, intensification may decrease the CWM mean pollen quality; consequently, intensification should indirectly select for pollinators with a low pollen-quality requirement [42,67]. 

However, it is difficult to define a quantitative pollinator matching trait that describes well the relationship between pollen quality and pollinator choice. We suggest that this trait may be defined by merging two fields of ecology that have rarely been combined: pollination and stoichiometric, the study of energy and the chemical–element balances of interacting organisms [68]. They can be combined by studying the degree of matching between pollen and pollinator stoichiometries [50]. Recent attempts to do so have been performed only for bees [69]. However, the homoeostasis capacity of pollinators may be high [70]. We suggest analysing differences in stoichiometry among pollinator taxa to help understand how intensification affects pollinator community composition.

## 4. Potential Effects of Intensification on Pollination Function on Grasslands

### 4.1. Grassland Intensification and Degree of Network Specialisation

One way to study the relationships between ecosystem functioning and plant–pollinator interactions is to analyse the latter’s degree of specialisation. Indeed, the more an interaction network is specialised, the higher the complementarity of its interactions and the differentiation of species niches [71]. An increase in complementarity implies that more functionally complementary species are needed to fulfil the ecosystem function [72]. Matching traits are useful for describing the niches of plants and pollinators [73] and providing mechanistic explanations for the degree of complementarity of plant–pollinator interactions. [74] showed that a plant community with higher floral diversity had higher plant–pollinator interaction network complementarity (measured by H2′, an index that describes the complementarity of interaction, [75]). In our review, we suggest that the CWM of nectar tube depth may decrease with intensification. Hence, flowers may be exploitable by a larger pool of pollinators, which reflects a plant community with more generalised exploitation-barrier traits. Moreover, intensification decreases forb richness [27] and thus may likely decrease flower functional diversity [76] due to its positive relationship with taxonomic diversity. Hence, intensification should generate networks with low functional complementary because of high niche overlap in floral traits among plant species. However, [25] found that intensification decreased plant species diversity but did not decreased H2′, which remained high overall. [77] observed the same lack of correlation without looking at the effect of intensification.

The degree of network specialisation may be explained in part by the matching traits but also by other processes, such as resource competition between pollinators. Hence, two competing pollinators with the same matching traits values may lead to fidelity for a flower [78] that they match less well. This highlights the need to define specialisation of plant–pollinator interactions carefully [21]. However, on intensively managed grasslands, despite the loss of pollinator species, the stability of pollination function loss may increase, because pollinators are more interchangeable than on less intensive grasslands.

### 4.2. Grassland Intensification and Pollination Visitation Frequency

Pollination function can be approximated in different ways. While pollinator abundance and diversity form part of it, interaction frequency seems more accurate [79] even though it has been criticised [80]. Few studies have addressed the relationship between intensification and interaction frequency. [25] and [40] observed no relationship between them in the same exploratory biodiversity project, whereas [46] found that less intensive grasslands have higher interaction frequency; however, a continuous land-use intensity index was not used in this last study and the results were not significant for all pollinator groups. Ref. [57] did not find a relation between intensification and interaction frequency in a set of 16 permanent grasslands belonging to an intensification gradient. The use of matching traits suggests that intensification decreases a community’s attractiveness for pollinators because of decreases in the CWMs of the floral display (at least before mowing; [46]), flower height [18,76], depending on the region studied) and reward production [76] which are all positively correlated with interaction frequency [76,81].

Intensification is likely to decrease the flower functional diversity (e.g., flower colour FD in [34]). Two assumptions can be made concerning the relationship between the FD of floral traits and interaction frequency. First, this relationship may be negative because a higher FD may blur the visual signal, leading to an increase in search time (serial processing; [82]). This assumption was confirmed in the studies of [76] and [83], which recorded a low taxonomic diversity of pollinators with a few generalist pollinator species representing most of interactions. Secondly, we expected a positive relationship between the functional diversity of floral traits and interaction frequency due to a better distribution of pollinators and a greater complementarity of pollinator niches [72]. Ref. [57] confirmed this hypothesis on permanent grasslands with 247 pollinator species. The highly diverse pollinator community recorded in this study may have increased the interaction frequency and the complementary between pollinator niches. Hence, more studies are needed to understand how floral trait functional diversity affects interaction frequency, and to confront niche theory with cognitive ecology, as the latter is based mostly on experiments performed under non-natural conditions [84]. Lastly, to improve understanding of how niche complementarity shapes the relations between floral functional diversity and interaction frequency, studies that include functional indices on each component of functional diversity (e.g., functional evenness, functional richness, functional divergence; [17]), not aggregative indices like functional entropy, Ref. [85] are needed.

### 4.3. Grassland Intensification and Body Traits of Pollinators Influencing the Quality of Pollination Interactions

Besides interaction frequency, information about the quality of interactions is needed [14,30]. Quality per interaction is often measured as the quantity of pollen deposited by a pollinator during a single visit to a freshly opened flower. This seems to be positively correlated with pollinator hairiness [86,87]. However, these two studies only focused on three cultivated plants species with easy access to the reproductive organs. Ref. [88] showed that pollinators’ facial pollen load increased with facial area and hairiness on 127 bee and fly species and 36 wild plants. Ref. [40] found that intensification led to a decrease in the CWM of both relative hairiness and body size of pollinators. An increase in the relative abundance of Diptera, which are less hairy [40,88] and smaller than bees [39] and have different pollination behaviour [89] may explain this result. This shift in pollinator community highlights the need to consider the phylogenetic signals between pollinator effect traits such as hairiness, body size and behaviour, and their respective effects independently.

### 4.4. Factors That May Limit Predicted Changes in Plant and Pollinator Communities Due to Grassland Intensification

Some factors may limit generalisation of changes in floral traits caused by grassland intensification. For instance, pollination is not the only way for plants to reproduce. These alternatives include self-pollination, vegetative reproduction (e.g., under grazing, which favours stoloniferous plants) and mass effects, such as the transfer of seeds between grasslands by hay machinery [27]. These processes promote the coexistence of plant species [90] and may favour floral traits inconsistent with the generalisation of floral traits. Pollinator abundance and characteristics, such as body size are affected by factors besides local intensification, especially landscape structure [91]. For instance, pollinator abundance increases with the proportion of semi-natural habitats in landscapes [92]. Furthermore, the literature results do not always support a decrease in body size with local intensification, because large body size can increase dispersal ability and avoidance of low-quality grassland patches [64,93]. Hence, future studies are needed to disentangle the contrasting effects of local and landscape factors.

## 5. Conclusions

Grassland intensification on floral traits has a cascading effect on the matching traits of pollinators and likely leads to the selection of plant species with generalised floral traits while decreasing the production of floral rewards. A decrease in mouthparts length and body size, two correlated traits, is consistent with the above-mentioned changes in floral traits. Furthermore, shifts in the taxonomic composition of pollinator communities toward Diptera-dominated communities can also be explained by generalised floral traits and the decrease in rewards production. We advocate for more studies to examine relationships between pollinator community composition and intensification to determine if the increase in the relative abundance of Diptera because of intensification is a common pattern.

Second, the data on how grassland intensification affects quantitative floral and pollinator traits are lacking. Indeed, while some of the matching traits that explain plant–pollinator interactions are well known––e.g., flower colour and insects’ visual systems have been studied for more than 100 years [94]––others, like flower odours, have received little attention or remain to be studied because they belong to different ecological fields. In particular, the impact of grassland intensification on floral rewards quality has rarely been studied.

Overall, little is known about the effect of intensification on grassland pollination function despite its importance in the current global pollination crisis. Most studies reviewed here addressed this issue with a quantitative dimension by using interaction frequency as a proxy of pollination function. We highlighted possible relationships between intensification and several qualitative dimensions of plant–pollinator interactions by focusing on pollinator hairiness and body size. In addition, although intensification leads to decreased pollination function, it selects for generalised plant species, but plants with generalised floral traits may be less pollen-limited than those with specialised floral traits [22].

Lastly, while the landscape scale has been recognised elsewhere as a main driver of plant–pollinator interactions [91], we showed that local factors may also change them drastically, despite having little knowledge about the ecosystem scale. This review places these gaps of knowledge into a clear framework, which we hope will motivate researchers to study them, especially because a holistic view of the human impact on pollination function and pollinators is needed to understand the current global pollination and pollinator crisis.

## Figures and Tables

**Table 1:** Summary of known and theoretical effects of agricultural intensification on plant-pollinator matching traits. A negative effect is indicated by a -; a positive effect by a +. The level of knowledge about these effects can be: tested in the literature (T), not tested in the literature (NT), indirect (I) or direct (D).

Matching Traits Categories	Matching Traits	Function	Agricultural Practices or Land-Use Index	Effect	Number of Grasslands	Knowledge Level	Country	References
**Signals**		Allow communication between plants and pollinators and thus interaction between them. Signals generate sensory experiences for pollinators that are different from an animal species to another						[31,32]
	Colour (hue)	Detection from background [33]	LUI	Shift toward white	69	T—D	Germany	[34]
	Photoreceptors and visual system	Matching level between visual system and colour	LUI	-	119	NT—I	Germany	[35]
	VOC emitted	Detection of flower [31]	Grazing and fertilization	None	2	T—D	France	[36]
	ND	Odour preferences	Not tested	ND		NT—I		
**Exploitation Barrier**		Prohibit interaction with a pollinator if its own matching traits are not adapted						[37]
	Nectar tube depth	Threshold to be reached by pollinator mouthpart length [38]	LUI	-	40	NT—D	Germany	[24,39]
	Relative proboscis length	Depth of exploitable flowers	LUI	-	40	T—D	Germany	[40]
**Rewards**		Essential food for pollinators. They gather mainly nectar as source of carbohydrates and pollen as source of proteins. Rewards are linked with pollinator matching traits which inform for instance on their food needs						[41,42,43]
	Nectar production	Total quantity of sugar in a grassland [28]	Nitrogen deposition	-	768	T—I	Great-Britain	[44]
			Livestock Unit/ha/year	-	561	T—D	Scotland	[45]
	Pollen production	Total quantity of pollen in a grassland	LUI	-	119	T—I	Germany	[25,46]
			Livestock Unit/ha/year	-	561	T—D	Scotland	[45]
	Body size	Quantity of pollinator food needs	LUI	-	40	T—D	Germany	[40]
	Phenology	Temporal availability of rewards [47]	Mowing, grazing, fertilization	(i.e., advances) or none	33	T—D	France	[18]
			Livestock Unit/ha/year		561	T—D	Scotland	[45]
	Sociability level	Duration of the breeding period	Not tested	-		NT—I		
	Nectar sugar concentration and nectar viscosity	Nectar feeding rate [48]	Not tested	+		NT—I		
	Anatomy of mouthpart	Adaptation to liquid viscosity	LUI	Shift toward sponging-sucking	40	NT—I	Germany	[40,49]
	Pollen amino acid concentration and protein content	Pollen quality [50]	LUI	-	40	NT—I	Germany	[39]
	Pollinator stoichiometric niche	Quality of pollinator food needs	Not tested	-		NT—I		

## Data Availability

The data presented in this study are available on request from the corresponding author.
